# Development of Piezoelectric Silk Sensors Doped with Graphene for Biosensing by Near-Field Electrospinning

**DOI:** 10.3390/s22239131

**Published:** 2022-11-24

**Authors:** Ming-Chan Lee, Guan-Ying Lin, Zheng-Yu Hoe, Cheng-Tang Pan

**Affiliations:** 1 Department of Mechanical and Electro-Mechanical Engineering, National Sun Yat-sen University, Kaohsiung 804, Taiwan; 2 Department of Physical Medicine and Rehabilitation, Kaohsiung Veterans General Hospital, Kaohsiung 813, Taiwan; 3 Institute of Advanced Semiconductor Packaging and Testing, College of Semiconductor and Advanced Technology Research, National Sun Yat-sen University, Kaohsiung 804, Taiwan

**Keywords:** piezoelectric, near-field electrospinning, PVDF, graphene, swallowing

## Abstract

A novel piezoelectric fiber sensor based on polyvinylidene fluoride piezoelectric (PVDF) doped with graphene is presented. The near-field electrospinning technology was used for developing the sensor. The uniform experimental design method was introduced to determine the ranges of experimental parameters, including the applied voltage, the drum speed range, the graphene doping ratios from 0% to 11 wt% in PVDF solution, and the electrode gap. By experimental results, the conductivities of PVDF solutions with different doping ratios of graphene increased from 19.6 μS/cm to 115.8 μS/cm. Tapping tests were performed to measure the voltages and currents produced by the piezoelectric fibers. The maximum output voltage was 4.56 V at 5 wt% graphene doping ratio in PVDF fibers, which was 11.54 times that of the pure PVDF sensors. Moreover, mechanical properties of the proposed sensor were measured. Motion intention and swallowing test, such as saliva-swallowing and eating, were carried out. When the subject spoke normally, the output voltage of the sensor was between 0.2 and 0.4 V, approximately. Furthermore, when the subject drank water and ate food, the output voltage of the sensor was between 0.5 and 1 V, approximately. The proposed sensor could be used to detect signals of the human body and serve as a wearable device, allowing for more diagnosis and medical treatment.

## 1. Introduction

In the past years, sensor systems have become important parts of our daily life. Sensors can be wearable devices and used for many applications, such as motion intention detection, real-time signal measurement, and a medical aid system. With peripherals, sensor systems can measure, detect, record and even analyze further health conditions of humans, which can also help more accurate diagnoses. Several types of sensors have been developed, including capacitive sensors [1,2,3,4], piezoresistive sensors [5,6], and piezoelectric sensors [7,8]. The linearity between the input and output, the hysteresis effect of signal feedback, and temperature sensitivity are important specifications regarding the performance of sensors. Furthermore, the size of the sensing area and the sensing range of pressure have been improved by previous studies [9]. The piezoelectric materials can be categorized into the main items: thin films, single crystals, ceramic, compound, and polymer.

Among the piezoelectric materials, Polyvinylidene fluoride (PVDF), zinc oxide, lead zirconate titanate (PZT) and aluminum nitride have high mutual conversion efficiency between electrical energy and mechanical energy. These piezoelectric materials are very sensitive to any small amount of strain, giving them excellent applicability in sensors. Although zinc oxide and aluminum nitride are easy to make into micro-nano-scale thin-film, the poor ductility and low piezoelectric coefficient of the materials will cause deflection and long-term deformation, making their application in the energy-harvesting sector very limited. PZT material has the advantages of good piezoelectric properties, but the material is brittle and cannot be used for long-term deformation. Furthermore, PZT material contains heavy-metal toxicity (lead), which is harmful to the human body. PVDF is a potential piezoelectric material in polymers. PVDF has the following advantages: light weight, low price, flexibility, decent piezoelectric coefficient, high response frequency and no elements of pollution by heavy metals, making it be selected as one of the main materials in this study.

Graphene, achieved by delamination of layered graphene, was developed at the University of Manchester in 2004. It was exfoliated from high-regularity, high-temperature pyrolysis graphite [10], and it was found in subsequent experiments that the single-layer graphene has excellent electrical conductivity. Single-layer graphene is a six-ring plane graphite structure and carbon atoms are composed of $sp^{2}$ chains. In appearance, the side length of single-layer graphene is about 2 to 5 μm and the thickness is 1 nm. It can be found that carbon nanocapsule (CNC), carbon nanotube (CNT) and monolayer graphene are all composed of carbon atoms linked by $sp^{2}$, in a 0–2 dimensional structure. However, the aspect ratios, surface-to-volume ratios, and the shapes among them are very different. Owing to the excellent electrical conductivity of graphene, it can be mixed with polymer materials to form composite materials and applied to electronic components such as supercapacitors [11,12], electromagnetic interference shielding (EMI) [13], dye-sensitized solar cells [14] and flexible optoelectronic films [15].

Electrospinning technology is an unsophisticated way to transform polymer materials into nano-scale fibers [16]. When the strength of the electric field increases, the electrostatic forces on the original surface overcome the surface tension, and the stretched polymer solution is ejected from the conical tip to form micron- to nanometer-slender fibers on the collector [17,18]. However, for PVDF fibers produced by traditional far-field electrospinning, the distance between the needle and the collecting plate is usually more than 10 cm. The polymer solution, which is easily affected by the electric field, will randomly spray the fibers, which will be deposited on the collection plate without order. This phenomenon weakens the piezoelectricity of the fiber. In addition, to spin out the fibers, a high voltage (hundreds of thousands of volts) is required [19,20].

Baumgarten et al. [21] presented the electrospinning process with methyl methacrylate (PMMA). The 1 µm width fiber was fabricated successfully. Reneker et al. [22] produced polymer fibers with diameters ranging from 40–2000 nm by electrospinning. These small fibers can support arrays of nanomachines and connect integrated arrays of nanomachines to larger-scale systems. Sun et al. [23] proposed the near-field electrospinning technology. It used 600 volts, and the distance between the needle and the collecting plate is only 500 µm during the electrospinning process. This technology successfully produces piezoelectric fibers in good order of 50–500 um in diameter and reduces the energy wasted.

Previous research presented the non-invasive measurement of the swallowing function with developed sensors or sensor-based monitoring during the swallowing events [24,25,26,27,28,29]. In this study, the proposed sensor was developed by the near-field electrospinning technique. The piezoelectric fiber sensor was produced by adding graphene nanoparticles to the PVDF solution by the near-field electrospinning process with a cylinder collection to make the piezoelectric fibers orderly. The electrospinning process was performed by different weight percentages of additives and related experimental parameters by the uniform experimental design method. The finished sensor was packaged by the interdigitated electrode (IDT), PET substrate, and polydimethylsiloxane (PDMS).

## 2. Materials and Method

### 2.1. Preparation of PVDF/Graphene Solution

Different amounts of graphene added and different proportions of solutions during the preparation process will affect the piezoelectric properties of the piezoelectric fibers after the near-field electrospinning process. Moreover, they affect conductivity, material stress and other properties of the sensor. The materials required for the preparation of PVDF solution and PVDF/graphene composite solution are shown in Table 1. The main material of the PVDF solution is polyvinylidene fluoride powder (Mw = 534,000).

The preparation of PVDF/graphene mixed solution was mainly divided into two parts: solution A and solution B. The proportion of the preparation is shown in Table 2. For solution A, a medicine spoon was used to weigh 0.9 g of PVDF powder and pour the powder into a scintillation vial to confirm that it was evenly distributed at the bottom of the scintillation vial. Then, 2.5 g of acetone was added by a syringe. Solution A was prepared after being stirred fully and evenly by a magnetic stirrer at a rotational speed of 400 rpm for 30 min at 25 °C. Solution B was prepared by mixing 0.2 g of surfactant and 0.5 g of dimethyl sulfoxide (DMSO). Exactly as with solution A, solution B was stirred by a magnetic stirrer at a rotational speed of 400 rpm for 30 min at 25 °C. Then, solution A was poured into solution B and stirred at a rotational speed of 400 rpm for 30 min at 25 °C. Finally, the mixed solution was left to stand for 30 min at room temperature to reduce the air bubbles. At this stage, the pure PVDF solution was completed.

**Figure 1:**
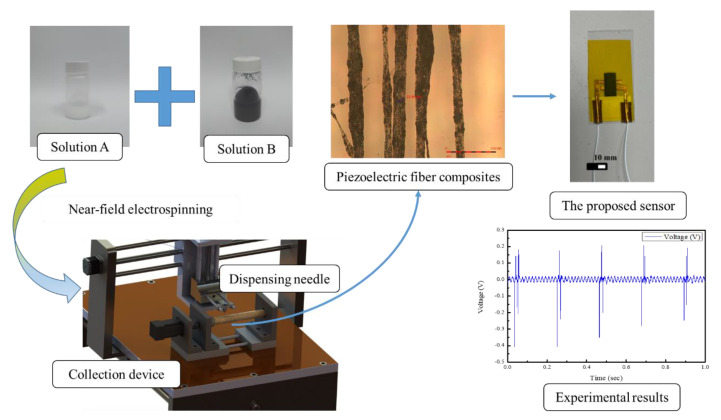
Schematic diagram of the procedure for the fabrication of PVDF/graphene sensor.

For the PVDF/graphene mixed solution, the procedure was similar to the preparation of the pure PVDF solution, but the required amount of graphene needed to be added into prepared solution B. Since the specific weight of solution A was heavier than that of solution B, solution A was poured into solution B. It was better to mix the solution fully and evenly. Like the pure PVDF preparation of solution, the PVDF/graphene mixed solution was left to stand for 30 min at room temperature to reduce the air bubbles. The experimental procedure for the fabrication of the proposed sensor is shown in Figure 1.

### 2.2. Uniform Experimental Design

The uniform experimental design can reduce experiment time while maintaining high quality and valid experiment results. The Kriging model depends on mathematical and statistical methods, satisfying an interpolation condition at every data point used to construct them. The Kriging model has more accurate function values throughout the trust region than the linear fit method.

**Figure 2:**
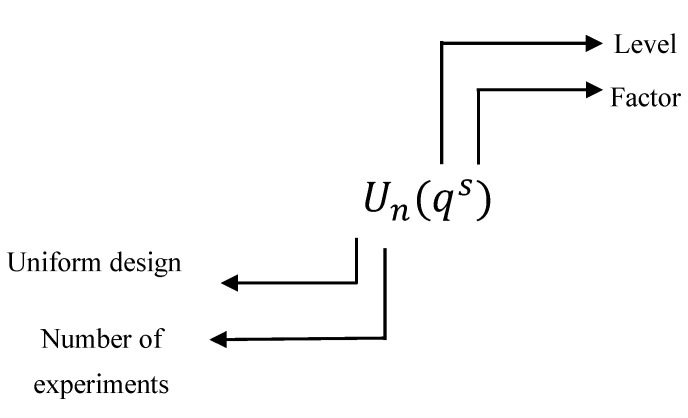
Parameters in the uniform experimental design method.

As shown in Figure 2, U is the symbol representing the uniform experimental design method, n represents the number of experiments to be performed, q represents the number of factor levels, and s represents the number of factors. There is an additional D value in each uniform experimental design table, which indicates the uniformity deviation value of the table. The D value represents the pros and cons of the uniformity of the table. The smaller value of D indicates the better uniformity.

With a specification of $U_{12}{}^{}\left(12^{10}\right)$, the four factors are the voltage of the near-field electrospinning, rotational speed of the drum collector, the weight percentage of graphene in the PVDF solution, and the gap between the sensor electrodes. The selected factors and their maximum and minimum values are shown in Table 3. To determine the ranges of experimental parameters, the voltage range was from 11 kV to 16.5 kV, the drum speed range was from 400 rpm to 950 rpm, and the graphene doping ratios from 0% to 11 wt% in PVDF solution, to find the optimized parameters by experiments. The output voltage of the sensor usually rises as the voltage of the near-field electrospinning is tuned larger and the gap between the sensor electrodes becomes smaller, but an appropriate parameter could be found. The uniform experimental design table used in this experiment is shown in Table 4.

.

### 2.3. Near-Field Electrostatic Spinning Process

**Figure 3:**
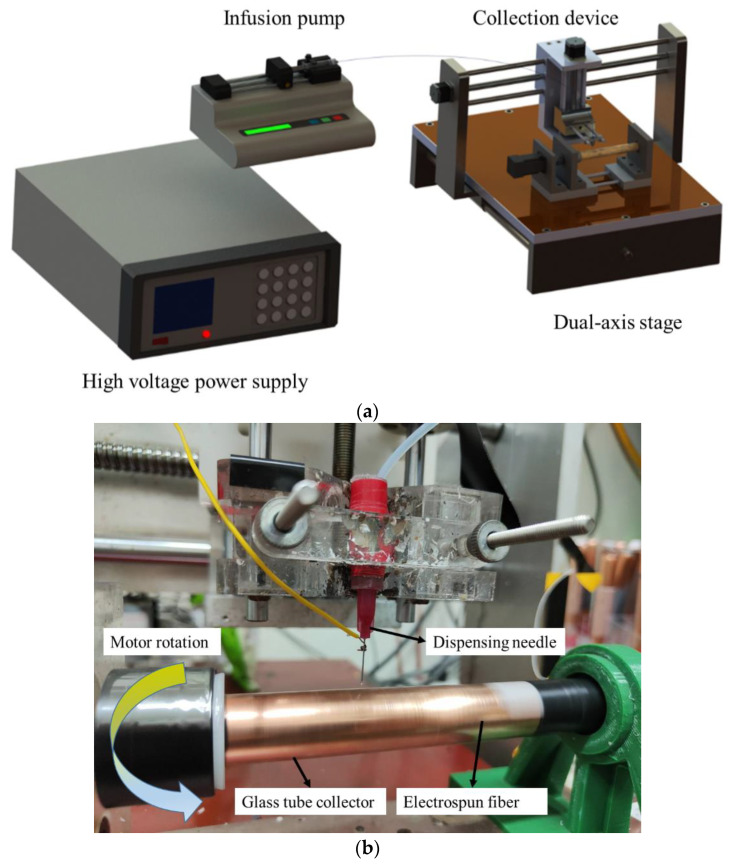
Equipment of the near-field electrospinning (**a**) Schematic diagram (**b**) Actual picture of the speed-adjustable drum collector.

Owing to the disordered and irregularly arranged fiber structures, the further application of the fibers obtained by the traditional electrospinning is limited. Compared with the fibers from the traditional electrospinning, the advantages of the fibers obtained by the near-field electrospinning include the large surface-to-volume ratio, smaller diameter, and aligned dipoles. Furthermore, the near-field electrospinning fabricates fibers at room temperature. Experiments can be conducted conveniently and flexibly. The main experimental equipment of the near-field electrospinning, as shown in Figure 3a,b, includes an XY dual-axis precision control stage, infusion pump, speed-adjustable drum collector, and high-voltage power supply. Supplemented with a copper-foil glass tube for fiber collecting and a replaceable industrial dispensing tip needle, the near-field electrospinning process performs and fibers can be collected.

**Figure 4:**
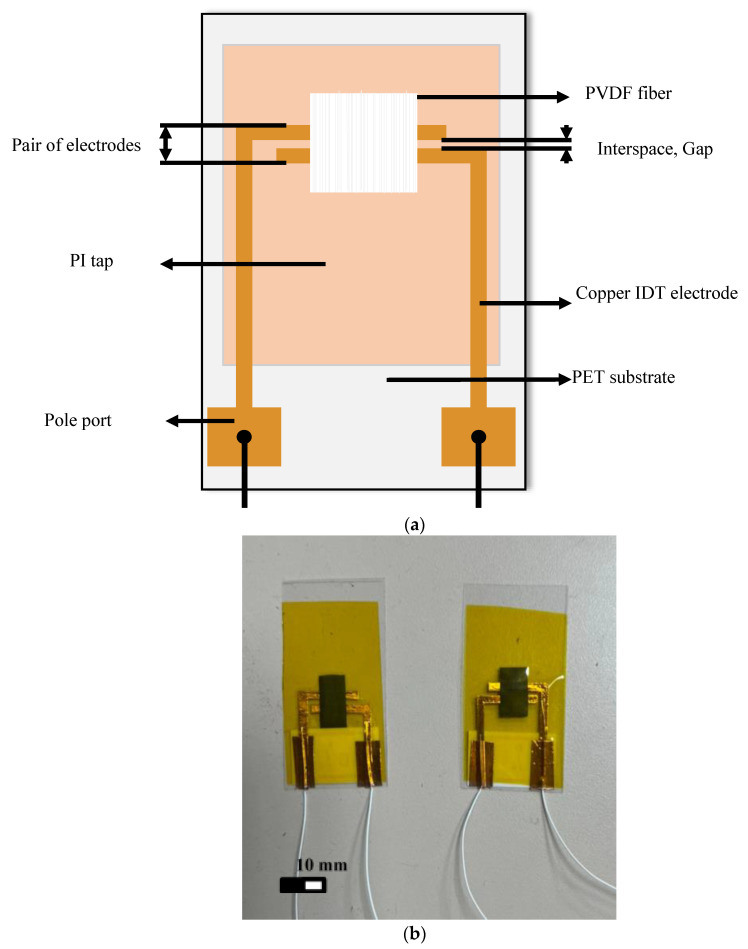
The packaged piezoelectric fiber sensor. (**a**) Schematic diagram (**b**) Actual picture.

First, the mixed solution was poured into the syringe and put on the infusion pump. The industrial dispensing needle was connected on the other end of the syringe. The positive high-voltage power supply generated a high-voltage electric field as a nozzle for ejecting the electrospinning fibers. The negative pole of the electric field was connected to the adjustable speed drum collector, copper-foil glass tube (the tube wall thickness was 0.5 mm, the outer diameter of the glass tube was 20 mm, and the copper-foil thickness was about 0.10 mm), and a negative high-voltage power supply. When the positive high-voltage power supply was applied to the needle of an industrial dispensing needle and the negative high-voltage power supply was applied to the drum collector simultaneously, a strong electric field was formed between the needle and the copper foil glass tube, causing the solution at the top of the needle to be affected by the additional electric field. When the strength of the electric field reached a value at which the repulsive force between the charges of the polymer solutions overcame the surface tension, the polymer solution in the original hemispherical shape elongated to form a cone, called a Taylor cone [30]. Meanwhile, the industrial dispensing head needle was controlled to move back and forth by the dual-axis precision control stage. The tachometer was used to measure and adjust the speed-adjustable drum collector. Ordered piezoelectric fibers with large area could be collected, and the dipole moments in the fibers showed regular arrangements parallel to the electric field along with the polarization direction. Finally, the packaged sensor, as shown in Figure 4a,b, was prepared with electrodes, polyethylene terephthalate (PET) film, and polyimide PI tapes.

### 2.4. Electrical Measurements

**Figure 5:**
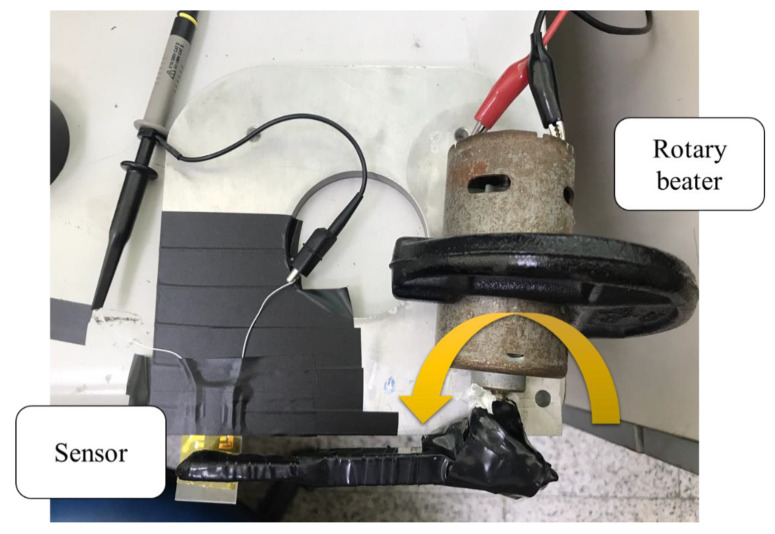
Top view of the electrical measurement device.

As shown in Figure 5, the sensor was tapped by a rotary beater and connected to the CHI-611D (CH Instruments, Inc., Austin, TX, USA) measuring instrument and the oscilloscope to measure the electrical signals. Since the piezoelectric fibers were deformed when they were beaten by the rotary beater, the piezoelectric response was generated. The CHI-611D measuring instrument was responsible for the measurement of the micro-current generated by the sensor due to tapping.

### 2.5. Mechanical Properties Measurements

When the piezoelectric material is subjected to a stress, the material deforms and its polarization density changes, as does its piezoelectric performance. The instrument used in this experiment is the AGS-50KNXD (Shimadzu, Japan) tensile testing machine. The length of the sample to be measured is 50 mm. By measuring the load and displacement of the sample, the stress-strain diagram and the Young’s coefficient are obtained.

## 3. Results and Discussion

### 3.1. Near-Field Electrospinning Fiber-Making Results

**Figure 6:**
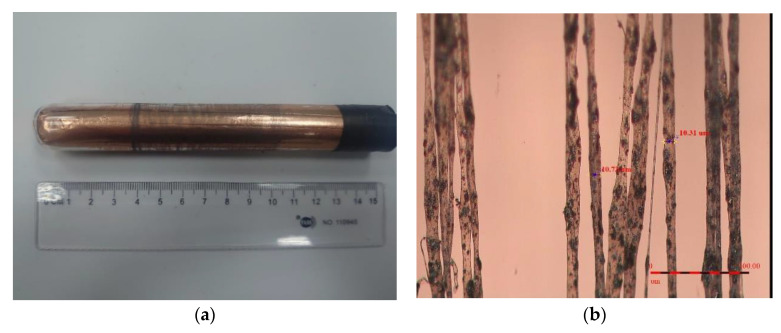
Electrospinning results of 1 wt% graphene in the PVDF mixed solution (**a**) Fibers collected by the tube (**b**) Orderly fibers.

**Figure 7:**
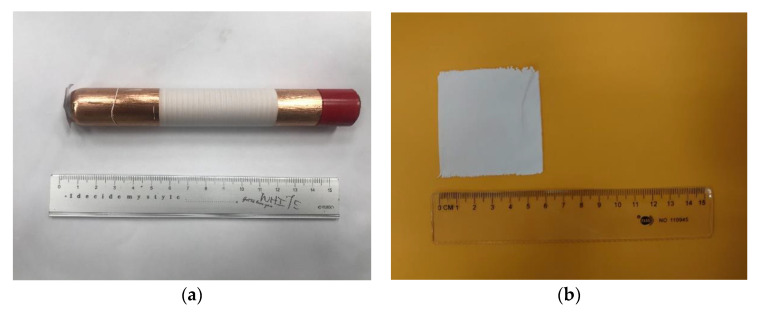
Electrospinning results of the pure PVDF fibers (**a**) Fibers collected by the tube (**b**) Pure PVDF fibers.

**Figure 8:**
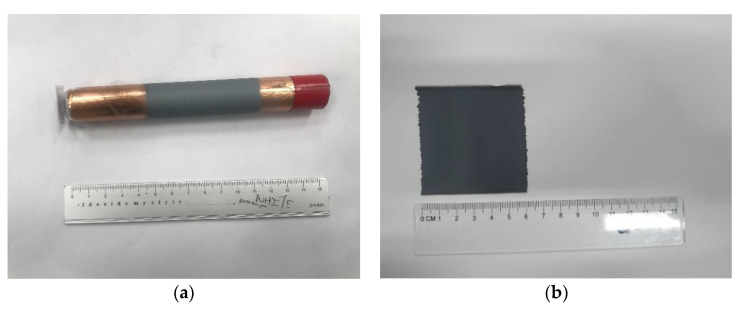
Electrospinning results of the PVDF fibers with graphene (**a**) Fibers collected by the tube (**b**) PVDF fibers with graphene.

Based on the uniform experimental design method, the experimental parameters were determined. The voltage ranges of the electric field were from 11 kV to 16.5 kV, and the rotational speed of the drum collector was from 400 rpm to 950 rpm. The weight percentage of graphene in the PVDF mixed solution was from 0% to 11%. The type of industrial dispensing needle used in the experiment was 25 G (aperture: 0.261 mm). The distance between the needle and the drum collector was 1 mm, and the movement speed of the dual-axis precision control stage was 2 mm/s. The infusion pump was used to control the propulsion flow rate of the solution at 20 mL/h so that the fibers could be produced smoothly. The fibers of 1 wt% graphene in the PVDF mixed solution were shown in Figure 6a,b for the preliminary test. The results of pure PVDF piezoelectric fibers were obtained as shown in Figure 7a,b, and the PVDF piezoelectric fibers doped with graphene were shown in Figure 8a,b. The electrospinning process continued for 30 min.

### 3.2. Conductivity Analysis of PVDF/Graphene Solution

In the electrospinning process, the solution of electrospinning will make the droplets of the solution break through the surface tension to form a Taylor cone. When the concentration of the solution is too high, the droplets will be discontinuous. However, the solution resulted in insufficient viscosity and it was easy to form excessively large droplets.

**Figure 9:**
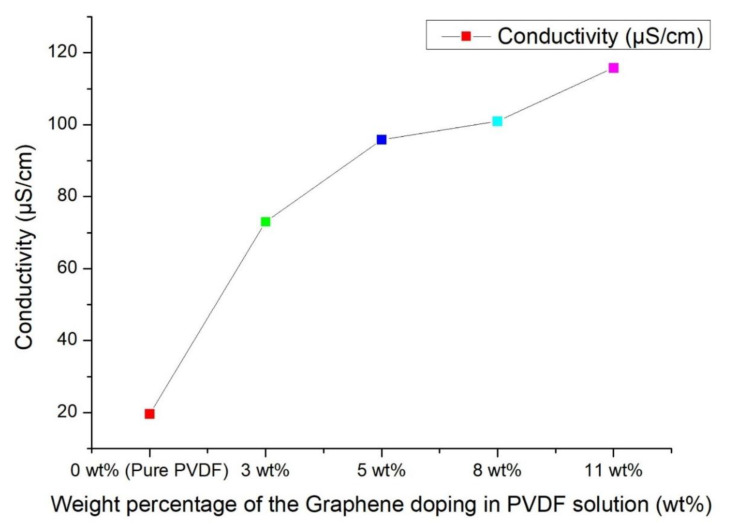
Conductivities with different weight percentages of the graphene doping in the PVDF solution.

By mixing the graphene powders in the solution, the conductivity of the solution rose. The conductivities of 0 wt%, 3 wt%, 5 wt%, 8 wt% and 11 wt% of PVDF/graphene mixed solution were measured. The results showed that the conductivity of 11 wt% PVDF/graphene mixed solution is 115.3 Μs/cm, which is the highest conductivity among sample solutions. The higher conductivity meant that it was easier to accumulate charges on the droplets, so the droplets broke through the surface tension and formed electrospinning fibers much more easily. Moreover, as shown in Figure 9, the conductivity of PVDF/graphene mixed solution was increased from 19.6 Μs/cm to 115.3 Μs/cm by adding graphene powder. The conductivity of the 11 wt% PVDF/graphene mixed solution increased about five times over that of the pure PVDF solution.

### 3.3. Piezoelectric Properties of Piezoelectric Fibers Using Uniform Experimental Design Method

**Figure 10:**
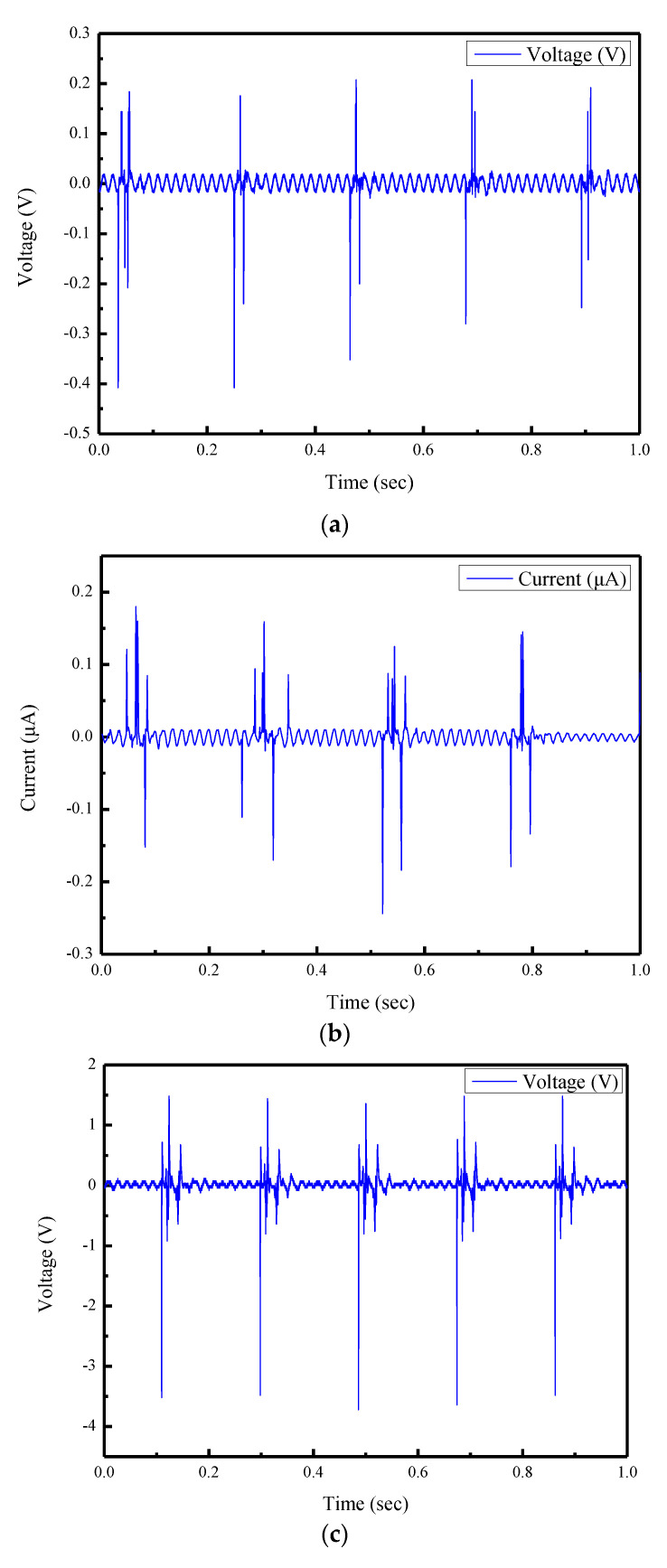

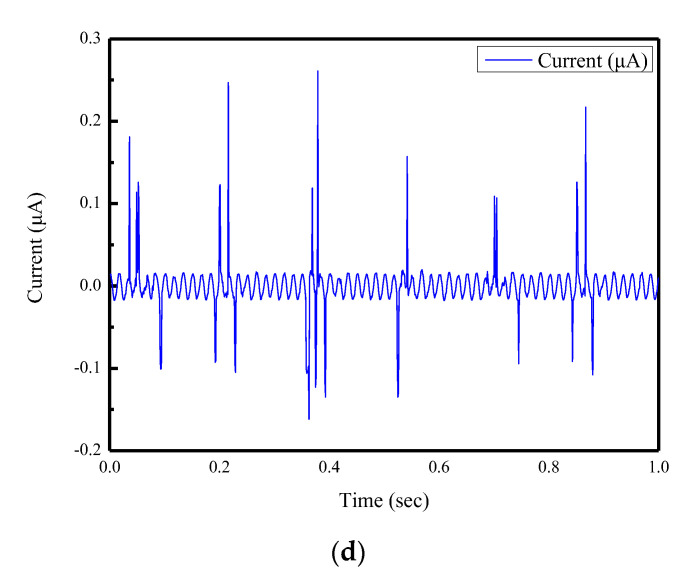
Results of the piezoelectric properties (**a**) Output voltage of pure PVDF piezoelectric sensor (**b**) Output current of pure PVDF piezoelectric sensor (**c**) Output voltage of 7 wt% PVDF/graphene piezoelectric sensor (**d**) Output current of 7 wt% PVDF/graphene piezoelectric sensor.

The piezoelectric properties between pure PVDF sensor and PVDF/graphene piezoelectric sensor were discussed. Sensors were tested by tapping tests with a rotary beater. As shown in Figure 10a–d, the maximum output voltage of the pure PVDF piezoelectric sensor was 0.395 V. The 7 wt% PVDF/graphene piezoelectric sensor had the maximum output voltage of all sensors; the output voltage was 3.672 V, which is about 9.3 times that of the pure PVDF sensor. The piezoelectric property of the fibers was improved by increases of the conductivity of the material.

### 3.4. Uniform Experimental Design Method Results

**Figure 11:**
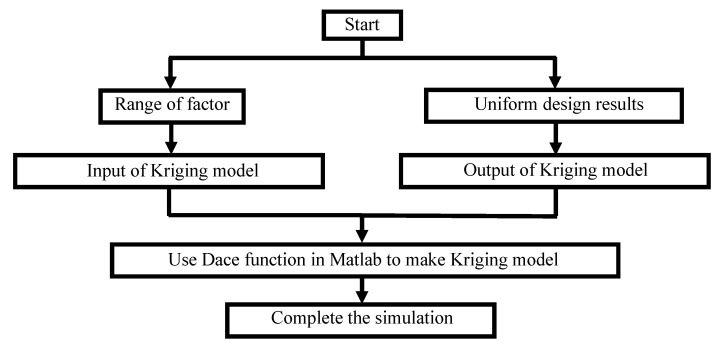
Flow chart to establish the Kriging model.

**Figure 12:**
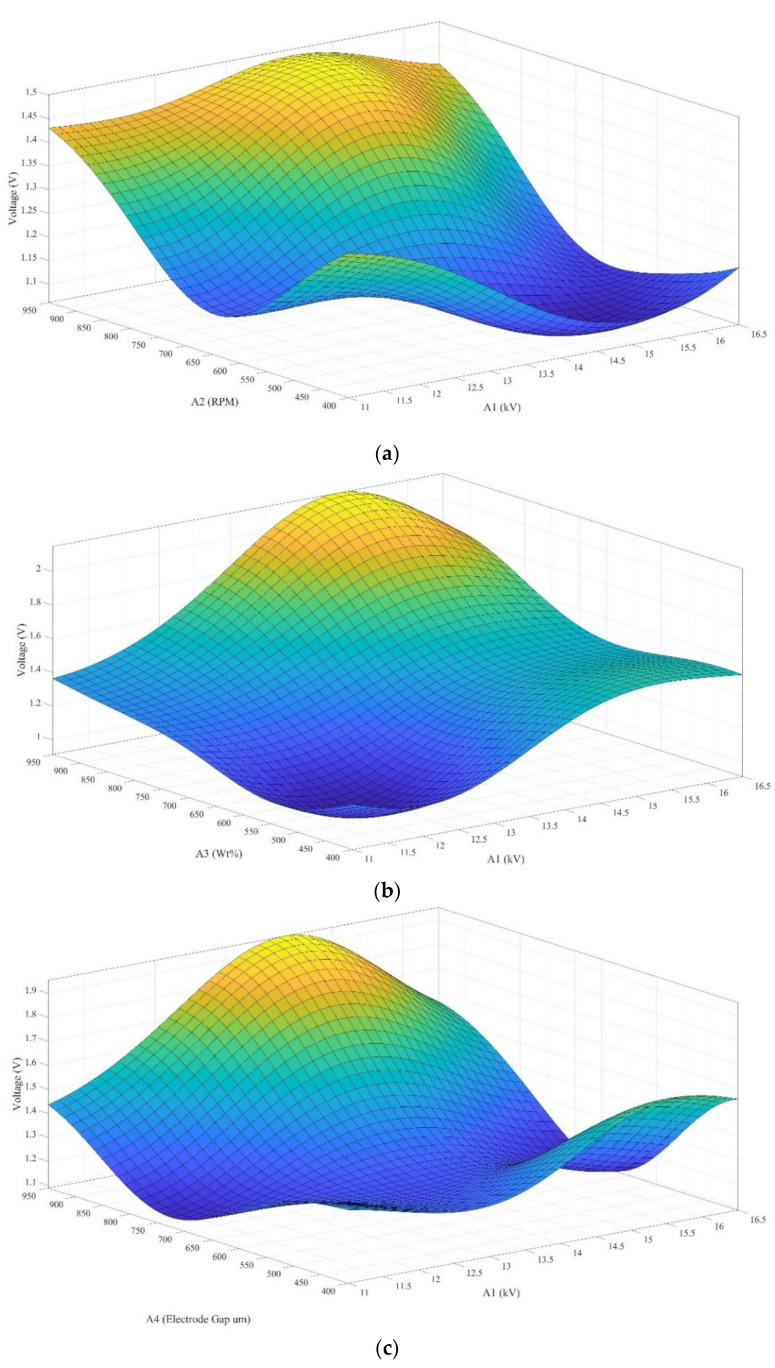

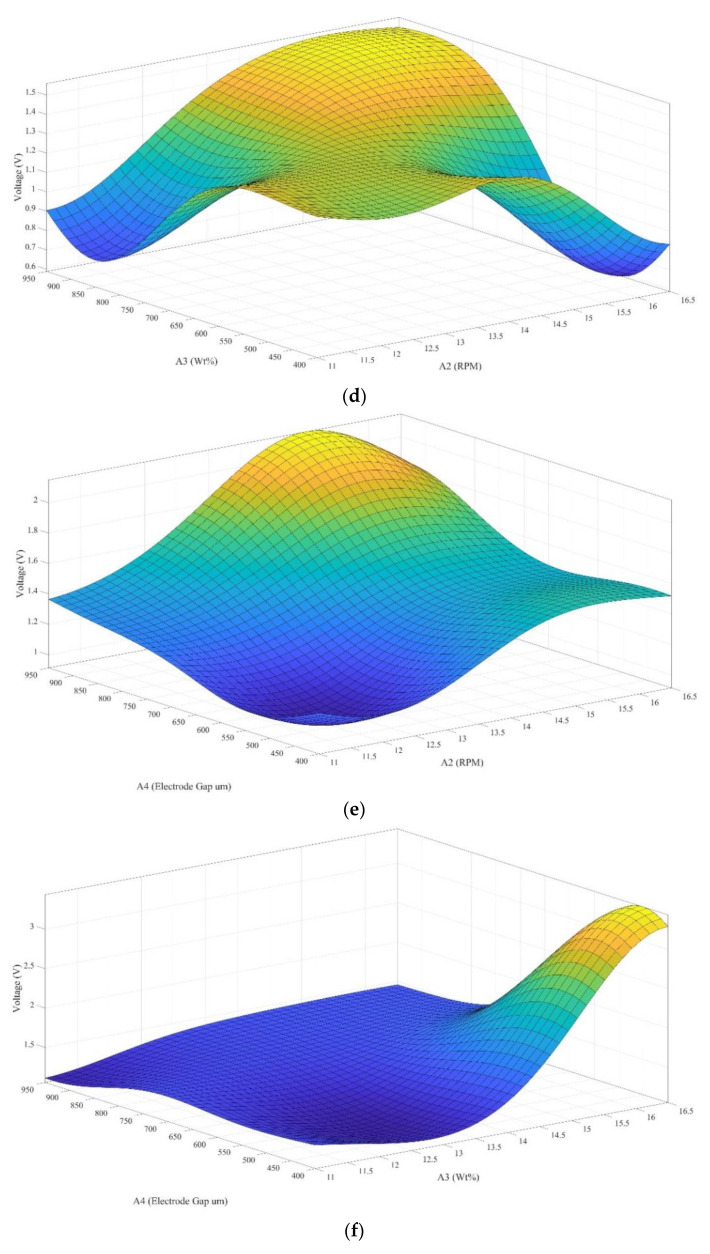
Simulation results of the Kriging model. (**a**) The relationship between the output voltage of the fiber, applied voltage in electrospinning, and the rotational speed of the drum collector. (**b**) The relationship between the output voltage of the fiber, applied voltage in electrospinning, and weight percentage of graphene. (**c**) The relationship between the output voltage of the fiber, applied voltage in electrospinning, and sensor electrode gap. (**d**) The relationship between the output voltage of the fiber, rotational speed of the drum collector, and weight percentage of graphene. (**e**) The relationship between the output voltage of the fiber, rotational speed of the drum collector, and sensor electrode gap. (**f**) The relationship between the output voltage of the fiber, weight percentage of graphene, and sensor electrode gap.

To interpret the relationship of each control factor in the uniform experimental design method in this study, the response surface of the Kriging model was used to depict four factors influencing the fiber. One of the codes is shown in Table 5. The normalization factor (between 0 and 1) established by these factors was used as the inputs of the model. The Dace function in MATLAB software was used to establish the response surface of the Kriging model. The flow chart of the reaction surface is shown in Figure 11. The relationship between the four factors in Table 5 could be expressed as Figure 12a–f, where each graph represented the interactions of each factor. The highest point of the curved surface was the parameter to reach the best performance of the piezoelectric fiber.

**Figure 13:**
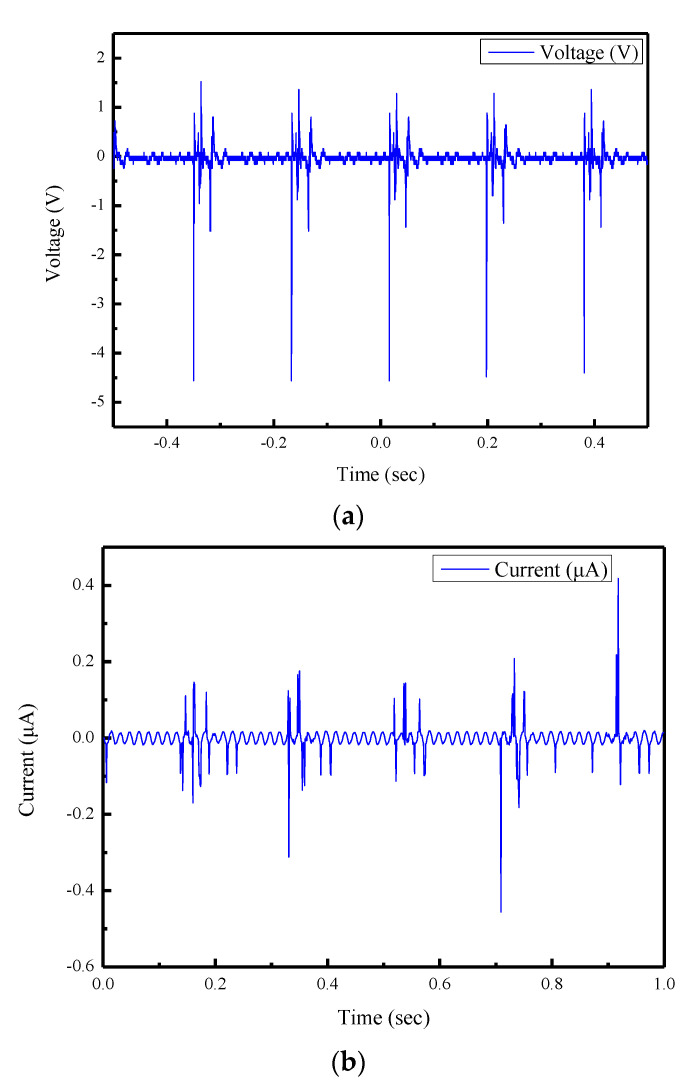
Experimental results of the 5 wt% doping ratio of graphene in PVDF solution. (**a**) Output voltage. (**b**) Output current.

From the results of the Kriging model, the optimal parameters of the electrospinning, as shown in Table 6, could be obtained. After the fiber fabrication and tapping test, the 5 wt% doping ratio of graphene in PVDF solution had the maximum output voltage of 4.56 V and output current of 0.456 μA. As shown in Figure 13a,b, the output voltage of 5 wt% PVDF/graphene fibers was about 11.54 times that of the pure PVDF piezoelectric fibers.

### 3.5. Mechanical Property Analysis of the Proposed Sensor

**Figure 14:**
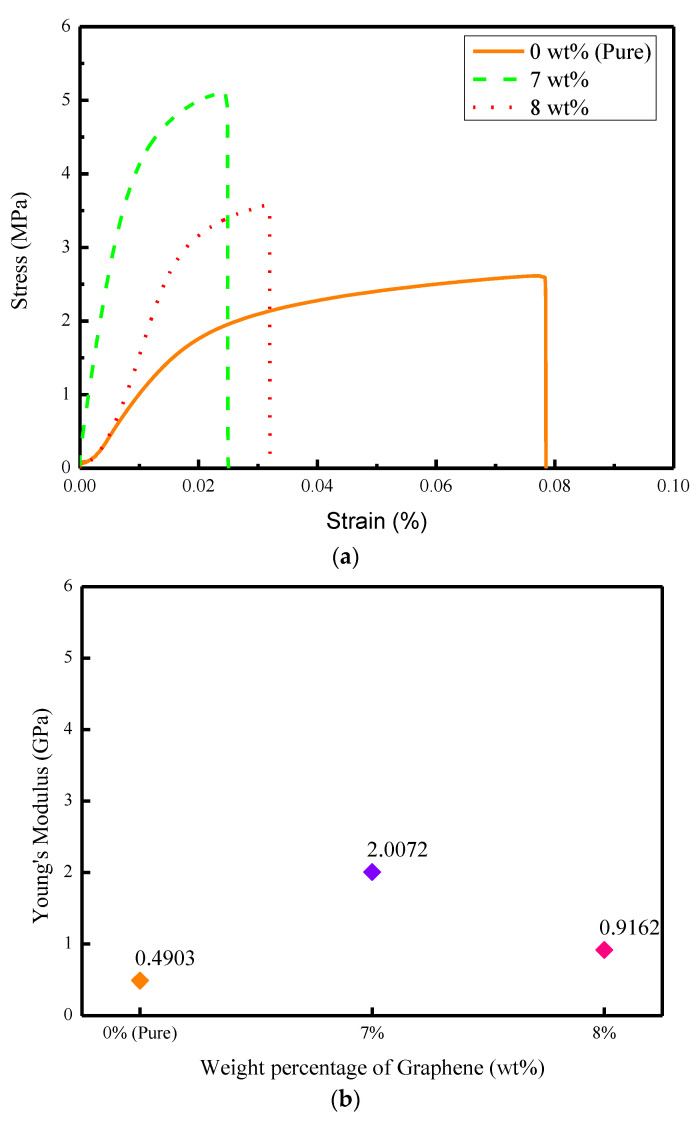

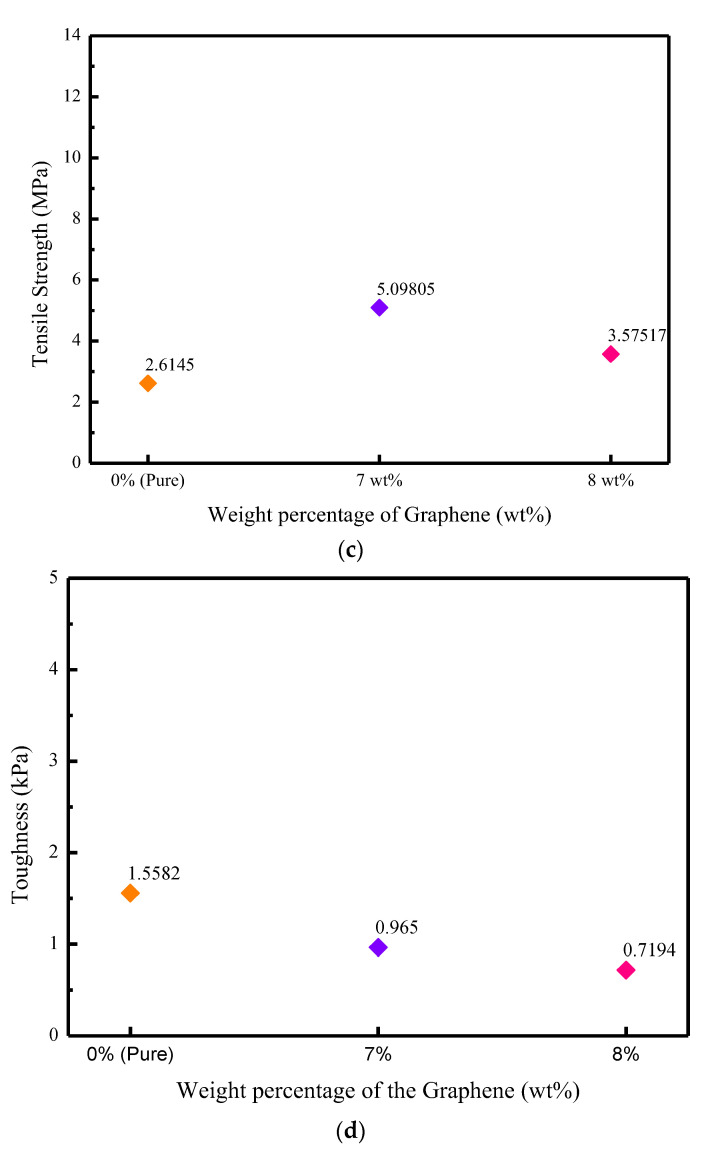
Experimental results of mechanical property tests. (**a**) Stress-strain curve. (**b**) Young’s modulus. (**c**) Tensile strength. (**d**) Toughness.

The experimental results of the tensile test (stress-strength curve), Young’s modulus, tensile strength, and toughness test between the pure PVDF fiber, 7 wt%, and 8% wt% PVDF/graphene fibers, are shown in Figure 14a–d. The PVDF/graphene fiber showed better performance in mechanical property tests. It also reflected that the PVDF/graphene fiber had good piezoelectric effect. It could be observed that the tensile strength of pure PVDF fiber was 2.6 MPa, and the tensile strengths of 7 wt% and 8 wt% PVDF/graphene fibers were 5.1 MPa and 3.6 MPa, respectively.

### 3.6. Applications of the Proposed Sensor

**Figure 15:**
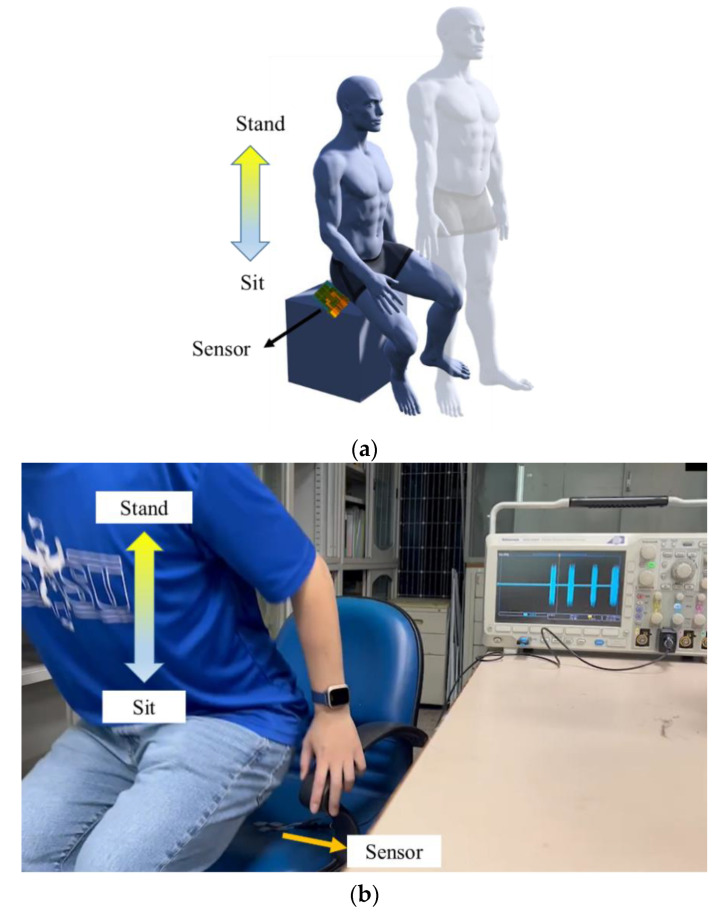
The motion intention test (**a**) Schematic diagram (**b**) Actual picture.

**Figure 16:**
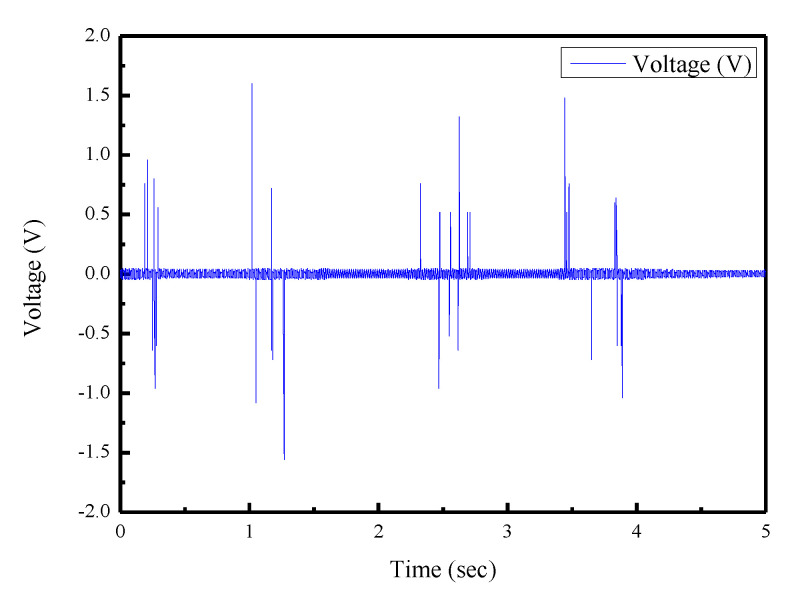
Experimental results of the stand–sit movement.

**Figure 17:**
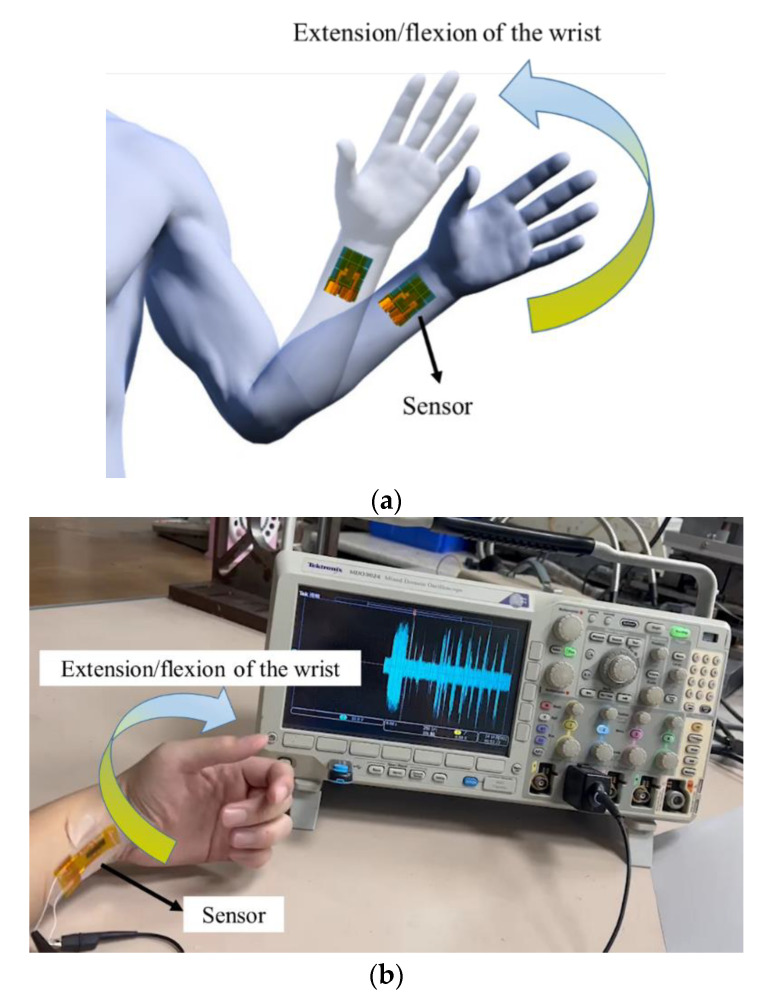
Extension/flexion of the wrist test. (**a**) Schematic diagram. (**b**) Actual picture.

**Figure 18:**
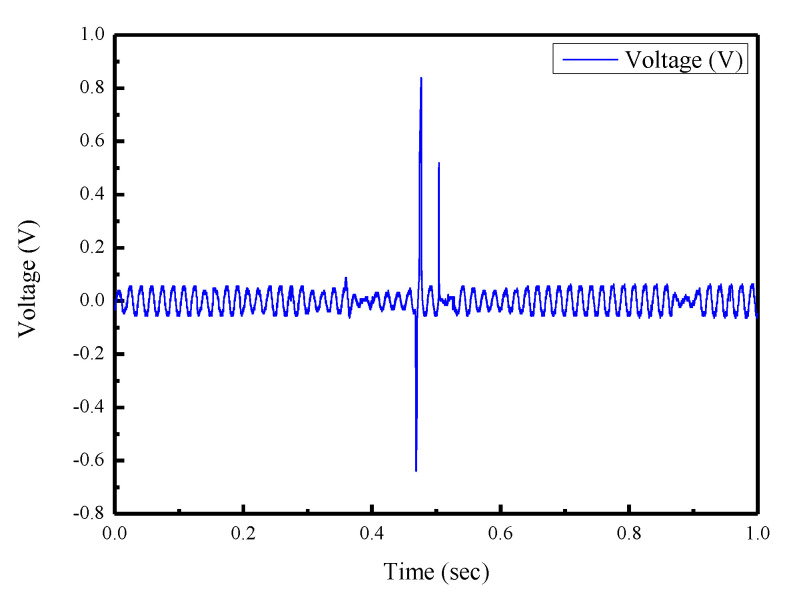
Experimental results of the wrist test.

The proposed sensor has potential for motion measurement of the human body and for developing a wearable sensing device. Therefore, preliminary tests of motion intention were performed. For the motion intention test, the sensor was attached to the chair and stand–sit movement cycles continued as shown in Figure 15a,b. Figure 16 shows the output voltages of the sensor. As shown in Figure 17a,b, the sensor was attached to the inner wrist (carpal joint) and wrapped on both sides with tapes. The wrist flexed and extended, and the output voltages were measured as shown in Figure 18.

From the experimental results, the maximum output voltage was 1.6 V when measuring the stand–sit movement of the human body. When measuring the extension and flexion of the wrist joint, the maximum voltage was 0.84 V. The experimental results showed that the proposed sensor has advantages of fit, flexibility, and elasticity, making it useful for more complex applications.

**Figure 19:**
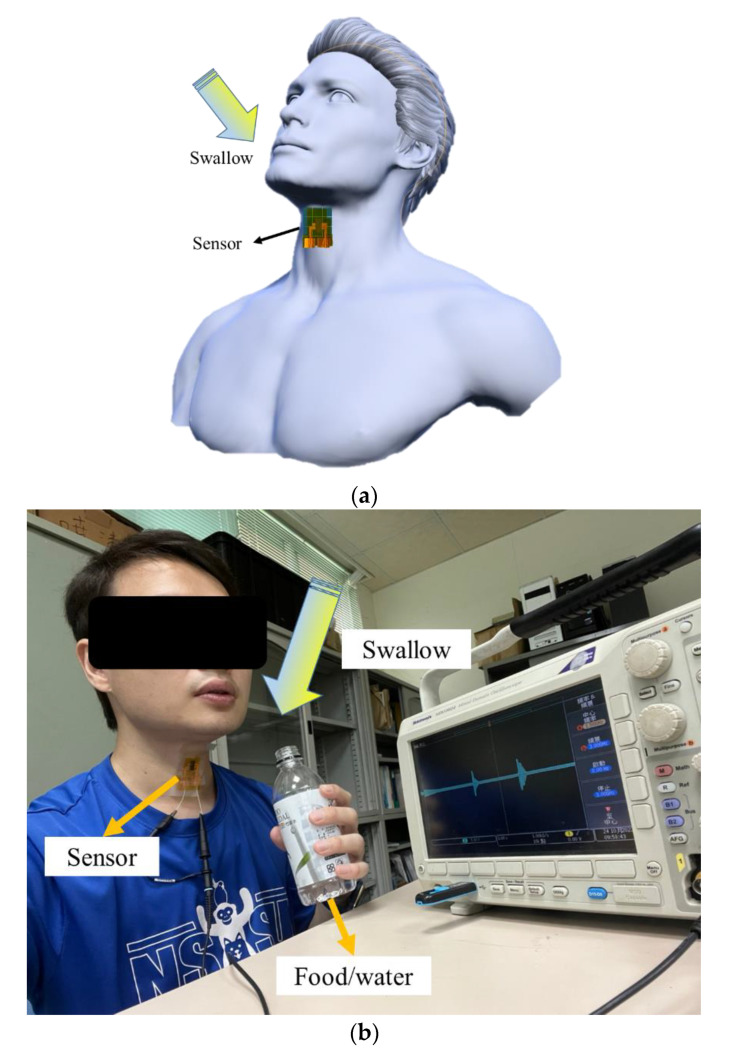
Swallowing test. (**a**) Schematic diagram. (**b**) Actual picture.

In the study, the proposed sensors were enhanced by the addition of graphene. Beside the motion measurement of the human body, the sensor was used to test and detect swallowing, allowing for a more accurate diagnosis and treatment. As shown in Figure 19a,b, the proposed sensor was attached to the skin surface of the laryngeal prominence and fixed by tapes. In the study, different swallowing and speaking were tested. Situations were considered as below:

1. Normal speaking: the subject spoke at about 150 words per minute.
2. Swallowing (saliva): the subject swallowed saliva deliberately.
3. Drinking: 30 mL water was drunk by the subject in each sip.
4. Eating food: the subject ate bread by an appropriate amount per bite.

**Figure 20:**
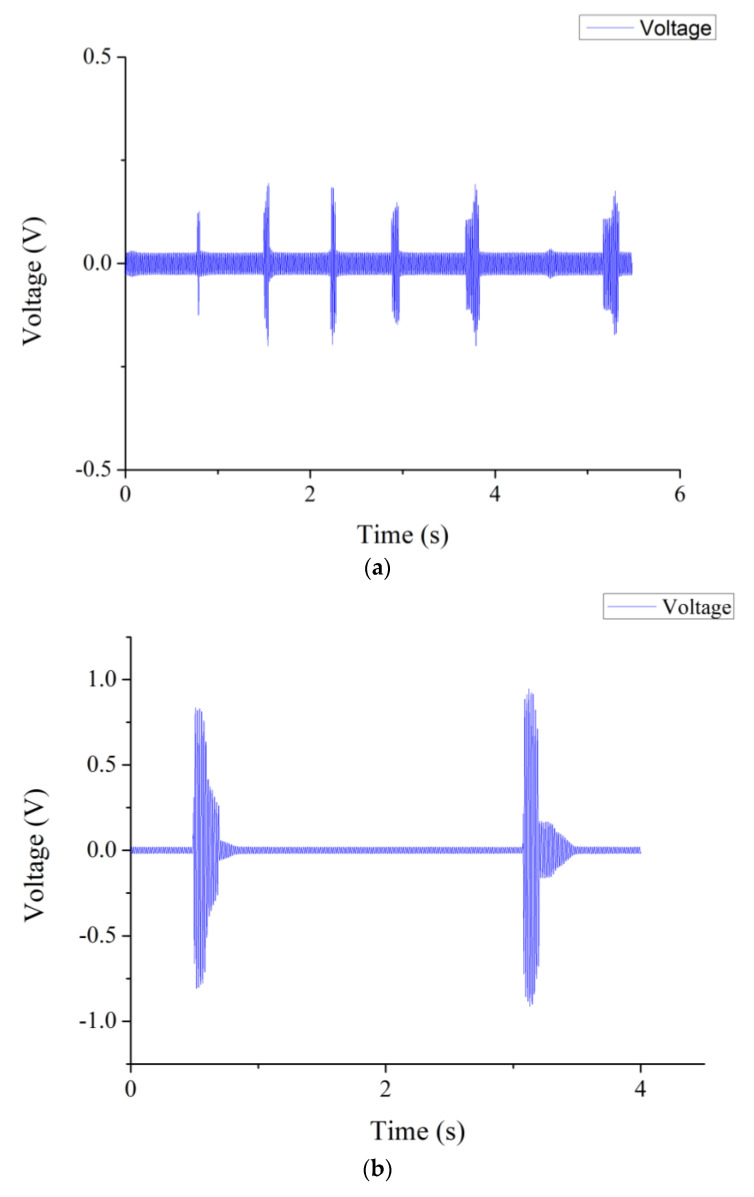

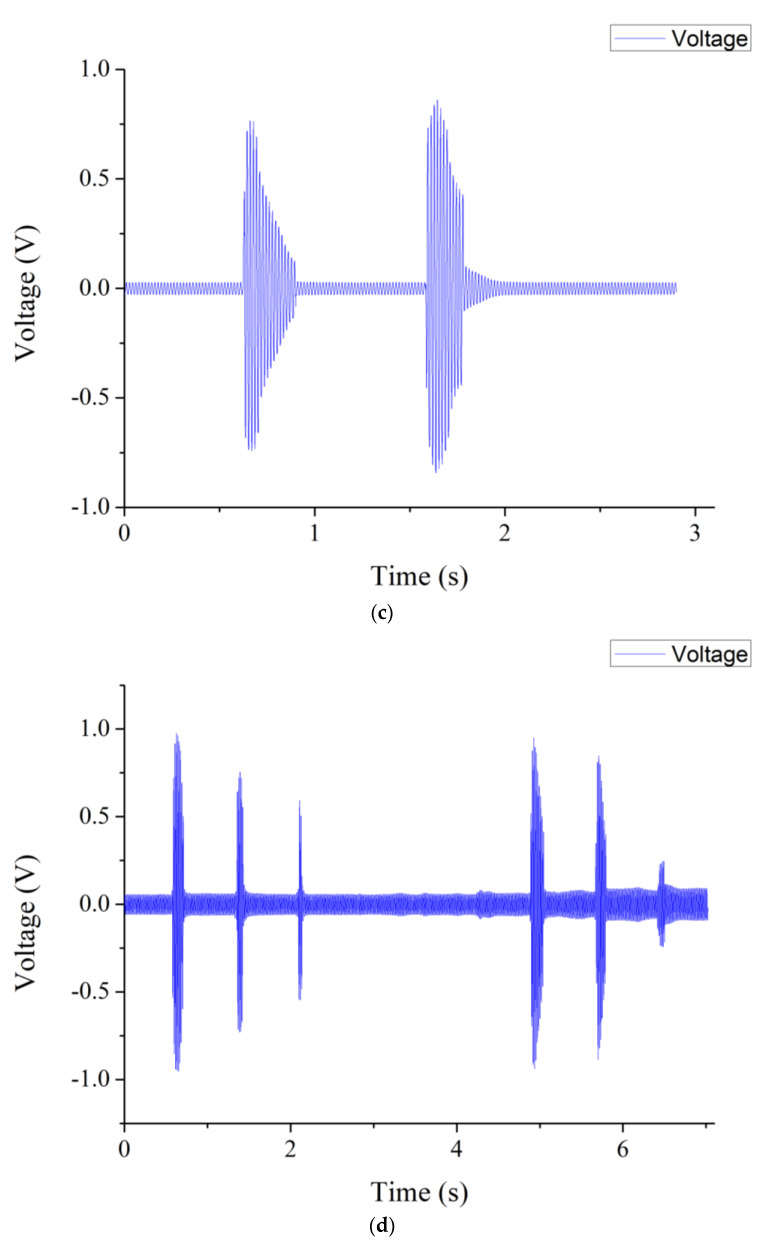
Experimental results of swallowing tests. (**a**) Normal speaking. (**b**) Swallowing. (**c**) Drinking. (**d**) Eating food.

When the subject spoke normally (at about 150 words per minute), the output voltage of the sensor was between 0.2 and 0.4 V approximately. Furthermore, when the subject drank water (50 mL each sip) and ate food (bread), the output voltage of the sensor was between 0.5 and 1 V, approximately. It could be observed that different swallowing behavior resulted in variant muscle contractions of the throat. Swallowing disorder (dysphagia) and swallowing dysfunction associated with muscle weakness due to natural aging could be detected by the proposed tactile sensor. The relationship between the muscle tremor of the throat and the output voltage of the proposed sensor was observed. The experimental results are shown in Figure 20a–d.

**Figure 21:**
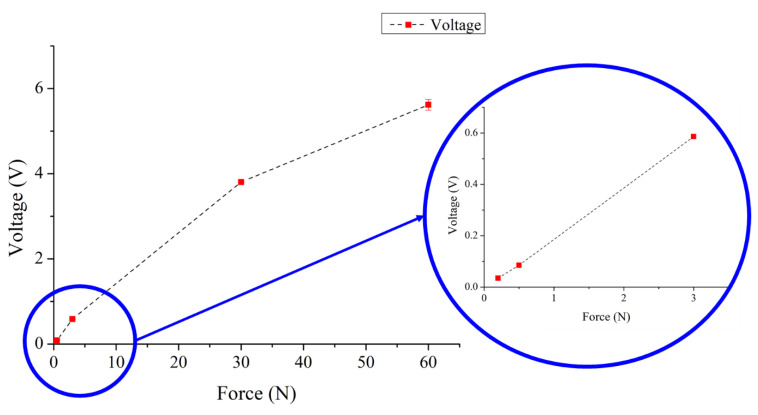
Output voltage–force curve of the proposed sensor.

**Figure 22:**
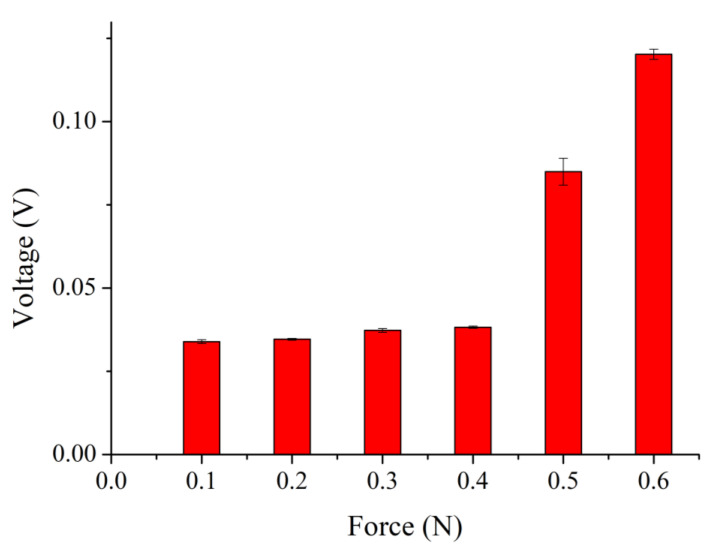
Relationship between output voltage and applied force within 0.6 N.

**Figure 23:**
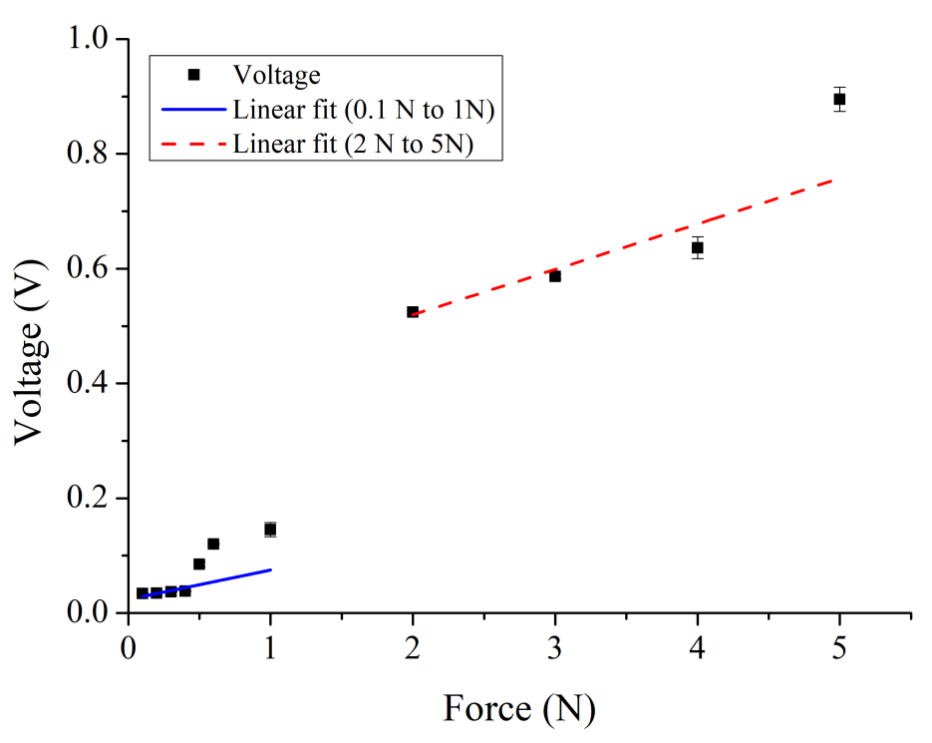
Ranges of linear regression.

The relationship between the output voltage of the proposed sensor and force was obtained by a force gauge HF-100 (Algol Instrument Co., Ltd., Taoyuan City, Taiwan). The range of the applied force was between 0.2–60 N. The range of corresponding output voltage was 0.03–5.62 V. The relationship between the applied force and the corresponding output voltage is shown in Figure 21. As the applied force was higher, the higher output voltage was achieved. As shown in Figure 22, the resolution of the proposed sensor was 0.1 N within 0.6 N. The range of corresponding output voltage was 0.03–0.12 V. Figure 23 shows the main ranges of linear regression. In the 0.1–1 N range, the sensitivity S = 0.05118 (V/N) and coefficient R^2^ = 0.14675. In the range 2–5 N, S = 0.07919 (V/N) and R^2^ = 0.79938. Characteristics of the proposed sensor are listed in Table 7.

## 4. Conclusions

In this study, a novel PVDF/graphene piezoelectric fiber sensor was developed using the near-field electrospinning technology. The applied voltage in electrospinning, rotational speed of the drum collector, weight percentage of graphene, and sensor electrode gap were process parameters. When the weight percentage of the graphene doping ratio was 11 wt%, the conductivity reached 115.3 μS/cm, which was 5.9 times that of the pure PVDF solution. From tapping tests, the output voltage of the 7 wt% doping ratio PVDF/graphene sensor was 3.672 V, which was 9.37 times that of the pure PVDF sensor, indicating that adding graphene to PVDF could improve the piezoelectric effect. Optimized by the uniform experimental design method and Kriging model, the optimal parameters were determined. The maximum output voltage was 4.56 V at 5 wt% graphene doping ratio in PVDF fibers, which was 11.54 times that of the pure PVDF sensors, indicating that the PVDF/graphene sensors were expected to provide a better piezoelectric effect. Through the mechanical property tests, it was observed that the tensile strengths of PVDF/graphene fibers at 7 wt% and 8 wt% doping ratio were 5.1 MPa and 3.6 MPa, which were larger than those of the pure PVDF fibers. Preliminary applications of the proposed sensor, such as the detections of the wrist extension/flexion and stand–sit movement intentions, were carried out. Furthermore, swallowing, such as saliva-swallowing, drinking and eating, were detected by the proposed sensor. The experimental results showed that the proposed sensor had the potential to be used to detect dysphagia and swallowing dysfunction. The PVDF/graphene piezoelectric fiber sensor made by the near-field electrospinning had the characteristics of ductility of PVDF polymer and piezoelectricity of graphene, which increases the possibility of practical applications.

## Figures and Tables

**Table 1:** Materials of the PVDF solution and PVDF/graphene mixed solution.

Ingredient	Chemical Formula	Appearance
PVDF	(C_12_H_13_NO_3_) n	White powder
DMSO	C_2_H_6_OS	Transparent liquid
Acetone	C_3_H_6_O	Transparent liquid
Graphene	C $sp^{2}$	Black powder
Surfactant	N/A	Brown paste

**Table 2:** PVDF/graphene mixed solution.

Solution A		Solution B		
PVDF (g)	Acetone (g)	DMSO (g)	Graphene (g)	Surfactant (g)
0.9	2.5	0.2	Uniform design	2.5

**Table 3:** Upper and lower boundaries of factors.

Factor	Applied Voltage (kV)	Rotational Speed of the Drum Collector (rpm)	Weight Percentage of Graphene (wt%)	Sensor Electrode Gap (mm)
Minimum	11 kV	400 rpm	0 wt%	1 mm
Maximum	16.5 kV	950 rpm	11 wt%	12 mm

**Table 4:** Uniform experimental design table $U_{12}{}^{}\left(12^{10}\right)$.

	Applied Voltage (kV)	Drum Collector Roll Rate (rpm)	Weight Percentage of Graphene (wt%)	Sensor Electrode Gap (mm)
$1{\mathrm{st}}^{}$ test	11	650	7	10
$2{\mathrm{nd}}^{}$ test	11.5	950	2	7
$3{\mathrm{rd}}^{}$ test	12	600	10	4
$4{\mathrm{th}}^{}$ test	12.5	900	5	1
$5{\mathrm{th}}^{}$ test	13	550	0	11
$6{\mathrm{th}}^{}$ test	13.5	850	8	8
$7{\mathrm{th}}^{}$ test	14	500	3	5
$8{\mathrm{th}}^{}$ test	14.5	800	11	2
$9{\mathrm{th}}^{}$ test	15	450	6	12
$10{\mathrm{th}}^{}$ test	15.5	750	1	9
$11{\mathrm{th}}^{}$ test	16	400	9	6
$12{\mathrm{th}}^{}$ test	16.5	700	4	3

**Table 5:** Experimental factors represented by each code of the Kriging model.

Factor	Applied Voltage (kV)	Rotational Speed of the Drum Collector (rpm)	Weight Percentage of Graphene (wt%)	Sensor Electrode Gap (mm)
Code Name	A1	A2	A3	A4

**Table 6:** Optimal parameters obtained by using Kriging reaction surface.

	Applied Voltage (kV)	Rotational Speed of the Drum Collector (rpm)	Weight Percentage of Graphene (wt%)	Sensor Electrode Gap (mm)
Optimal parameters	12.5	900	5	1

**Table 7:** Characteristics of the proposed sensor.

Resolution	Upper Force	Linear Range	0.1–1 N		2–5 N	
			Sensitivity	R^2^	Sensitivity	R^2^
0.1 (N)	60 (N)	0.1–1 (N)/2–5 (N)	0.05118 (V/N)	0.14675	0.07919 (V/N)	0.79938

## Data Availability

Not applicable.

## References

[1] Ma L., Shuai X., Hu Y., Liang X., Zhu P., Sun R., Wong C.-P. (2018). A highly sensitive and flexible capacitive pressure sensor based on a micro-arrayed polydimethylsiloxane dielectric layer. J. Mater. Chem. C.

[2] Kim S.-W., Oh G.-Y., Lee K.-I., Yang Y.-J., Ko J.-B., Kim Y.-W., Hong Y.-S. (2022). A Highly Sensitive and Flexible Capacitive Pressure Sensor Based on Alignment Airgap Dielectric. Sensors.

[3] Luo Y., Shao J., Chen S., Chen X., Tian H., Li X., Wang L., Wang D., Lu B. (2019). Flexible Capacitive Pressure Sensor Enhanced by Tilted Micropillar Arrays. ACS Appl. Mater. Interfaces.

[4] Pagoli A., Chapelle F., Corrales-Ramon J.-A., Mezouar Y., Lapusta Y. (2022). Large-Area and Low-Cost Force/Tactile Capacitive Sensor for Soft Robotic Applications. Sensors.

[5] Jeong H., Noh Y., Ko S.H., Lee D. (2019). Flexible resistive pressure sensor with silver nanowire networks embedded in polymer using natural formation of air gap. Compos. Sci. Technol..

[6] Kim K.-H., Hong S.K., Jang N.-S., Ha S.-H., Lee H.W., Kim J.-M. (2017). Wearable Resistive Pressure Sensor Based on Highly Flexible Carbon Composite Conductors with Irregular Surface Morphology. ACS Appl. Mater. Interfaces.

[7] Kim N.-I., Chang Y.-L., Chen J., Barbee T., Wang W., Kim J.-Y., Kwon M.-K., Shervin S., Moradnia M., Pouladi S. (2020). Piezoelectric pressure sensor based on flexible gallium nitride thin film for harsh-environment and high-temperature applications. Sens. Actuators A Phys..

[8] Yang Y., Pan H., Xie G., Jiang Y., Chen C., Su Y., Wang Y., Tai H. (2020). Flexible piezoelectric pressure sensor based on polydopamine- modified BaTiO3/PVDF composite film for human motion monitoring. Sens. Actuators A Phys..

[9] Abdul Razak A.H., Zayegh A., Begg R.K., Wahab Y. (2012). Foot Plantar Pressure Measurement System: A Review. Sensors.

[10] Novoselov K.S., Geim A.K., Morozov S.V., Jiang D., Zhang Y., Dubonos S.V., Grigorieva I.V., Firsov A.A. (2004). Electric field effect in atomically thin carbon films. Science.

[11] Wang H., Hao Q., Yang X., Lu L., Wang X. (2009). Graphene oxide doped polyaniline for supercapacitors. Electrochem. Commun..

[12] Yan J., Wei T., Shao B., Fan Z., Qian W., Zhang M., Wei F. (2010). Preparation of a graphene nanosheet/polyaniline composite with high specific capacitance. Carbon.

[13] Liang J., Wang Y., Huang Y., Ma Y., Liu Z., Cai J., Zhang C., Gao H., Chen Y. (2009). Electromagnetic interference shielding of graphene/epoxy composites. Carbon.

[14] Hong W., Xu Y., Lu G., Li C., Shi G. (2008). Transparent graphene/PEDOT–PSS composite films as counter electrodes of dye-sensitized solar cells. Electrochem. Commun..

[15] Geng X., Niu L., Xing Z., Song R., Liu G., Sun M., Cheng G., Zhong H., Liu Z., Zhang Z. (2010). Aqueous-Processable Noncovalent Chemically Converted Graphene–Quantum Dot Composites for Flexible and Transparent Optoelectronic Films. Adv. Mater..

[16] Huang Z.-M., Zhang Y.-Z., Kotaki M., Ramakrishna S. (2003). A review on polymer nanofibers by electrospinning and their applications in nanocomposites. Compos. Sci. Technol..

[17] Gong G., Wu J., Jiang L. (2011). Novel polyimide materials produced by electrospinning. Prog. Chem..

[18] Xu J., Abecassis M., Zhang Z., Guo P., Huang J., Ehmann K.F., Cao J. Accuracy Improvement of Nano-Fiber Deposition by Near-Field Electrospinning. Proceedings of the 9th International Workshop on Microfactories (IWMF 2014).

[19] Fuh Y.-K., Chen P.-C., Huang Z.-M., Ho H.-C. (2015). Self-powered sensing elements based on direct-write, highly flexible piezoelectric polymeric nano/microfibers. Nano Energy.

[20] Schiffman J.D., Schauer C.L. (2007). Cross-linking chitosan nanofibers. Biomacromolecules.

[21] Baumgarten P.K. (1971). Electrostatic spinning of acrylic microfibers. J. Colloid Interface Sci..

[22] Reneker D.H., Chun I. (1996). Nanometre diameter fibres of polymer, produced by electrospinning. Nanotechnology.

[23] Sun D., Chang C., Li S., Lin L. (2006). Near-field electrospinning. Nano Lett..

[24] Malvuccio C., Kamavuako E.N. (2022). The Effect of EMG Features on the Classification of Swallowing Events and the Estimation of Fluid Intake Volume. Sensors.

[25] Choi Y., Kim M., Lee B., Yang X., Kim J., Kwon D., Lee S.-E., Kim H., Nam S.I., Hong S. (2020). Development of an Ultrasonic Doppler Sensor-Based Swallowing Monitoring and Assessment System. Sensors.

[26] Shieh W.-Y., Wang C.-M., Cheng H.-Y.K., Wang C.-H. (2019). Using Wearable and Non-Invasive Sensors to Measure Swallowing Function: Detection, Verification, and Clinical Application. Sensors.

[27] Kurihara Y., Kaburagi T., Kumagai S., Matsumoto T. (2019). Development of Swallowing-Movement-Sensing Device and Swallowing-State-Estimation System. IEEE Sens. J..

[28] Maeda M., Kadokura M., Aoki R., Kawakami M., Koyama Y., Nishiyama M., Watanabe K. Non-invasive swallowing examination device using hetero-core fiber optic pressure sensor. Proceedings of the 2021 IEEE 3rd Global Conference on Life Sciences and Technologies (LifeTech).

[29] Koyama Y., Nishiyama M., Aoki R., Higashimori Y., Watanabe K. Swallowing Measurement for a Healthy Subject Using a Hetero-Core Optical Fiber Sensor. Proceedings of the 2019 IEEE 8th Global Conference on Consumer Electronics (GCCE).

[30] Taylor G.I. (1964). Disintegration of water drops in an electric field. Proc. R. Soc. Lond. Ser. A.

